# Supplementary material for: A sensitive MOKE and optical Hall effect technique at visible wavelengths: insights into the Gilbert damping
Source: Nat Commun. 2025 Jul 17;16:6423. doi: 10.1038/s41467-025-61249-4 (PMC12271360; doi:10.1038/s41467-025-61249-4)
Supplement: Supplementary file 1 — Supplementary Information [file 41467_2025_61249_MOESM1_ESM.pdf]

**Supplementary Information**

# A sensitive MOKE and optical Hall effect technique at visible wavelengths: insights into the Gilbert damping

Nadav Am-Shalom[1], Amit Rothschild[1], Nirel Bernstein[1], Michael Malka[1], Benjamin Assouline[1], Daniel Kaplan[2], Tobias Holder[2], Binghai Yan[2,3], Igor Rozhansky[4], *Amir Capua[1]

[1]Insititute of Electrical Engineering & Applied Physics, The Hebrew University of Jerusalem, Jerusalem 91904, Israel.
[2]Department of Condensed Matter Physics, Weizmann Institute of Science, Rehovot 7610001, Israel.
[3]Department of Physics, Pennsylvania State University, University Park 16802, Pennsylvania, USA.
[4]National Graphene Institute, University of Manchester, Manchester M13 9PL, United Kingdom.

*e-mail: amir.capua@mail.huji.ac.il

## Contents

Supplementary Note 1: Description of the setup using Jones calculus .......... 3
Supplementary Note 2: Measurements at the fundamental and second harmonics .......... 6
Supplementary Note 3: Deviations from ideal cross-polarization .......... 7
Supplementary Note 4: Detection using a balanced detector .......... 10
Supplementary Note 5: XRD, AFM, and resistivity characterization .......... 11
Supplementary Note 6: Shot noise analysis with non-ideal polarizers .......... 15
Supplementary Note 7: Considerations for measuring weak noise signals .. 17
Supplementary Note 8: Gilbert damping measurements .......... 18
Supplementary Note 9: Measurements at 638 nm .......... 22

## Supplementary Note 1: Description of the setup using Jones calculus

The Jones matrix calculus is beneficial for establishing the relationship between $V_{PD}$ and $\Phi_K$. We refer to the geometry of the experiment as illustrated schematically in Fig. S1 and follow the conventional formalism, e.g. as derived by Gomez et al. in Ref. [S1].

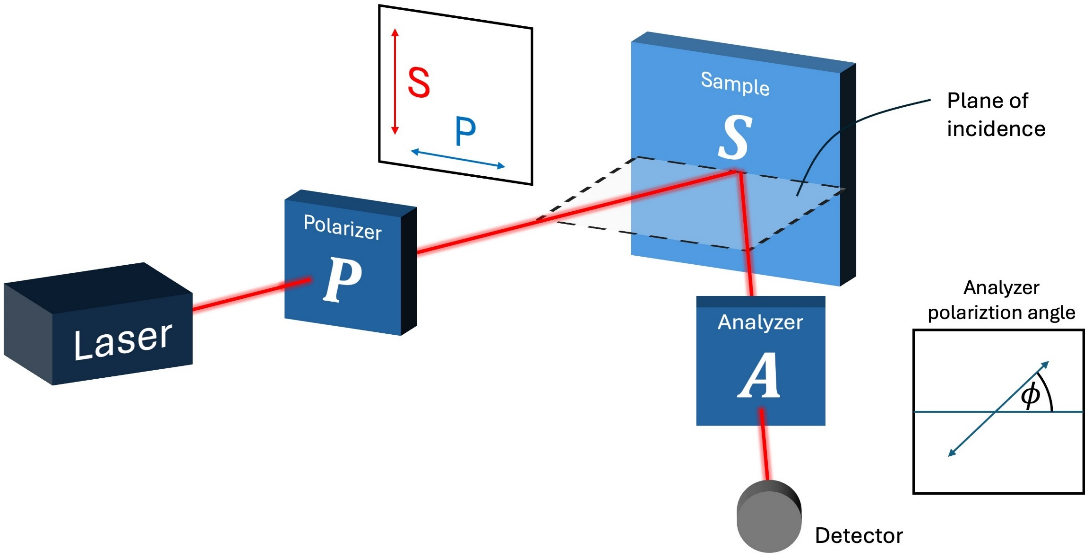


**Fig. S1. Schematic illustration of the experimental setup where the optical elements are expressed in Jones matrices calculus.**

In our setup, the polarizer and analyzer are cross-polarized for maximal extinction. Below we present the derivation for the general case of an analyzer having a transmission axis at an angle $\phi$ relative to plane of incidence. The emitted laser beam can be described by the generic Jones vector $\begin{bmatrix} E_p^i \\ E_s^i \end{bmatrix}$ where $E_p^i$ and $E_s^i$ are the amplitudes of the electrical field in the $p$ and $s$ polarizations, respectively. The beam first passes a linear polarizer having the Jones matrix $\boldsymbol{P}$, reflects off the sample with a reflection matrix $\boldsymbol{S}$ and then passes the analyzer described by the Jones matrix $\boldsymbol{A}$. Accordingly, $\boldsymbol{P}$ and $\boldsymbol{A}$ are given by:

$$\boldsymbol{P} = \begin{bmatrix} 0 & 0 \\ 0 & 1 \end{bmatrix}, \quad \boldsymbol{A} = [X \quad Y] \begin{bmatrix} \cos^2\phi & \sin\phi \cdot \cos\phi \\ \sin\phi \cdot \cos\phi & \sin^2\phi \end{bmatrix} = \begin{bmatrix} X\cos^2\phi + Y\sin\phi \cdot \cos\phi \\ Y\sin\phi \cdot \cos\phi + X\sin^2\phi \end{bmatrix},$$

where $\boldsymbol{S}$ describing the sample is given by:

$$\boldsymbol{S} = \begin{bmatrix} r_{pp} & r_{ps} \\ r_{sp} & r_{ss} \end{bmatrix}.$$

In $\boldsymbol{S}$, the complex diagonal elements, $r_{ss}$ and $r_{pp}$, are simply the Fresnel reflection coefficients for the $s$ and $p$ polarizations, respectively. The magneto-optical Kerr effect (MOKE), or alternatively, the optical Hall effect (OHE), are manifested by the off-diagonal elements, $r_{ps}$ and $r_{sp}$. As derived from the Lorentz-Drude model, $|r_{ps}|$ and $|r_{sp}|$are linear in the applied magnetic field, $B$. For simplicity, we use the general notation $B$ rather than $B_{AC}$ and $B_{DC}$. The electrical field reaching the detector is given by:

$$\begin{bmatrix} E_p^f \\ E_s^f \end{bmatrix} = \boldsymbol{A} \cdot \boldsymbol{S} \cdot \boldsymbol{P} \cdot \begin{bmatrix} E_p^i \\ E_s^i \end{bmatrix}.$$

The generated photocurrent is proportional to the optical intensity such that:

$$V_{PD} = \frac{1}{2} \cdot c\epsilon_0 \rho_0 \cdot \begin{bmatrix} E_p^{f^*} & E_s^{f^*} \end{bmatrix} \cdot \begin{bmatrix} E_p^f \\ E_s^f \end{bmatrix},$$

where $\rho_0$ is the optical responsivity, and $c$ and $\epsilon_0$ are the speed of light and permittivity in vacuum, respectively. Subsequently, we arrive to Eq. (1) of the manuscript:

$$V_{PD} = \frac{1}{2} \cdot c\epsilon_0 \rho_0 \left|E_s^i\right|^2 \cdot \left[\sin^2 \phi \, |r_{ss}|^2 + \cos^2 \phi \, \left|r_{ps}\right|^2 + 2 \cos \phi \sin \phi \cdot Re\{r_{ss} r_{ps}^*\}\right]. \quad (1)$$

In the perfect cross-polarization arrangement, $\phi = 0°$, and $V_{PD} = \frac{1}{2} \cdot c\epsilon_0 \rho_0 \cdot \left|E_s^i\right|^2 \left|r_{ps}\right|^2$. Therefore, $V_{PD} \propto B^2$ as seen in the manuscript. The complex Kerr angle $\Phi_K$ is defined by $\Phi_K = r_{ps}/r_{pp}$. Therefore, to extract $|\Phi_K|$, an additional measurement of $|r_{ss}|$ is required, by measuring the reflectance of the samples before the beam entered the analyzer.

In the measurements of $|r_{ss}|$, the laser beam was attenuated to prevent optical saturation. Additionally, a $100\,K\Omega$ load resistor was used to ensure that the bandwidth of the photodiode was well above the modulation frequency. In contrast, in the measurement of $|r_{ps}|$, the photodiode was connected directly to the lock-in amplifier having a $10\,M\Omega$ input impedance which was required to detect the small OHE signal. This led to a bandwidth of $\sim 400\,Hz$, slightly lower than $\omega_{mod}$ which was taken into account. The generated photocurrent $I_{PD}(t)$ can be expressed by:

$$I_{PD}^{ss}(t) = \eta I_{opt} L_{OD}^{ss} |r_{ss}|^2 \cdot \mathrm{rect}^2(t)$$

$$I_{PD}^{ps}(t) = \eta I_{opt} \left|r_{ps}\right|^2 (1 + \sin(\omega_{mod} t))^2$$

where the upper indices indicate the $r_{ps}$- and $r_{ss}$- related quantities, $\eta$ is the optical responsivity of the detector, $I_{opt}$ is the laser power, and $L_{OD}^{ss}$ and $\mathrm{rect}(t)$ are the optical attenuation and rectangular on-off modulation waveform, respectively, applied in the measurement of $|r_{ss}|$. The voltage signal at the input of the lock-in amplifier is given by $V_{sig}(t) = R_L \cdot I_{PD}(t)$ where $R_L$ is the load resistance. The recorded lock-in voltages following homodyne detection are given by $V_{PD}^0 = \eta I_{opt} R_L^{ss} L_{OD}^{ss} \mathcal{F}_{rect} |r_{ss}|^2$ and $V_{PD,B_{mod}} = \eta I_{opt} R_L^{ps} \mathcal{F}_{sine} L_{BW}^{ps} |r_{ps}|^2$ where $\mathcal{F}_{rect}$ and $\mathcal{F}_{sine}$ are the Fourier components at $\omega_{mod}$ of the rectangular and the all-positive sinusoidal waveforms, and $L_{BW}^{ps}$ is the attenuation resulting from the limited bandwidth in the measurement of $|r_{ps}|$. Finally, the magnitude of the complex Kerr angle is given by:

$$|\Phi_K| = \sqrt{\frac{V_{PD,B_{mod}}}{V_{PD}^0}} \cdot \sqrt{\frac{R_L^{ss}}{R_L^{ps}} \frac{L_{OD}^{ss}}{L_{BW}^{ps}} \frac{\mathcal{F}_{rect}}{\mathcal{F}_{sine}}}.$$

Table S1 compares the measured $|\Phi_K|$ values with those previously reported in the literature. The table presents the $|\Phi_K|$ values extrapolated to the maximal field applied in our experiments ($0.45\ T$). The values are in relatively good agreement with those reported in Refs. [S2-5]. The differences may be attributed to the different film growth conditions and the film thicknesses.

| Sample | Present work | Ref. [S3], Uba et al. (2017) Ref. [S2], Uba et al. (2000) | | Ref. [S4] Schnatterly et al. (1969) | | Ref. [S5] Stern et al. (1964) | |
|---|---|---|---|---|---|---|---|
| | Measured @ $0.45\ T$ $[mdeg]$ | Measured @ $1.5\ T$ $[mdeg]$ | Extrapolated to $0.45\ T$ $[mdeg]$ | Measured @ $1\ T$ $[mdeg]$ | Extrapolated to $0.45\ T$ $[mdeg]$ | Measured in units of $[mdeg/T]$ | Extrapolated to $0.45\ T$ $[mdeg]$ |
| Au | $\lvert\Phi_K\rvert = 0.39$ | $\lvert\Phi_K\rvert = 1.18$ | $\lvert\Phi_K\rvert = 0.33$ | $\lvert\Phi_K\rvert = 0.61$ | $\lvert\Phi_K\rvert = 0.27$ | - | - |
| Cu | $\lvert\Phi_K\rvert = 0.33$ | $\lvert\Phi_K\rvert = 0.84$ | $\lvert\Phi_K\rvert = 0.28$ | $\lvert\Phi_K\rvert = 0.66$ | $\lvert\Phi_K\rvert = 0.29$ | - | - |
| Pt | $\lvert\Phi_K\rvert = 0.47$ | $\lvert\Phi_K\rvert = 0.1$ | $\lvert\Phi_K\rvert = 0.3$ | - | - | - | - |
| Al | $\lvert\Phi_K\rvert = 0.22$ | - | - | - | - | $\theta_k = 0.4$ | $\theta_k = 0.18$ |
| Ta | $\lvert\Phi_K\rvert = 0.37$ | - | - | - | - | - | - |

**Table S1. Comparison of measured and previously reported $|\Phi_K|$ values at $440\ nm$.**

## Supplementary Note 2: Measurements at the fundamental and second harmonics

In the experimental setup, the external magnetic field $B_z$ reduces to zero between the magnets. Consequently, $r_{ps}^2 \propto (1+\sin(\omega_{mod}t))^2 = 1.5 + 2\sin(\omega_{mod}t) - 0.5\cos(2\omega_{mod}t)$ which is modulated at $\omega_{mod}$ and $2\omega_{mod}$. To experimentally validate these harmonic components, additional measurements were carried out on the Py film by setting the reference to $\omega_{mod}$ and $2\omega_{mod}$. These measurements are presented in Fig. S2. The figure presents the recorded data of both the "in-phase" $X$-channel and the "out-of-phase" $Y$-channel. It is seen that the response at $2\omega_{mod}$ also exhibits a quadratic dependence on $B_{AC}$ and that its amplitude is approximately one-quarter that of the signal at $\omega_{mod}$, as expected. Additionally, the signal at $2\omega_{mod}$ predominantly appears on the $Y$-channel, in contrast to the signal at $\omega_{mod}$ which is measured on the $X$-channel. This is consistent as well with the sine and cosine dependences appearing in the expression of $r_{ps}^2$.

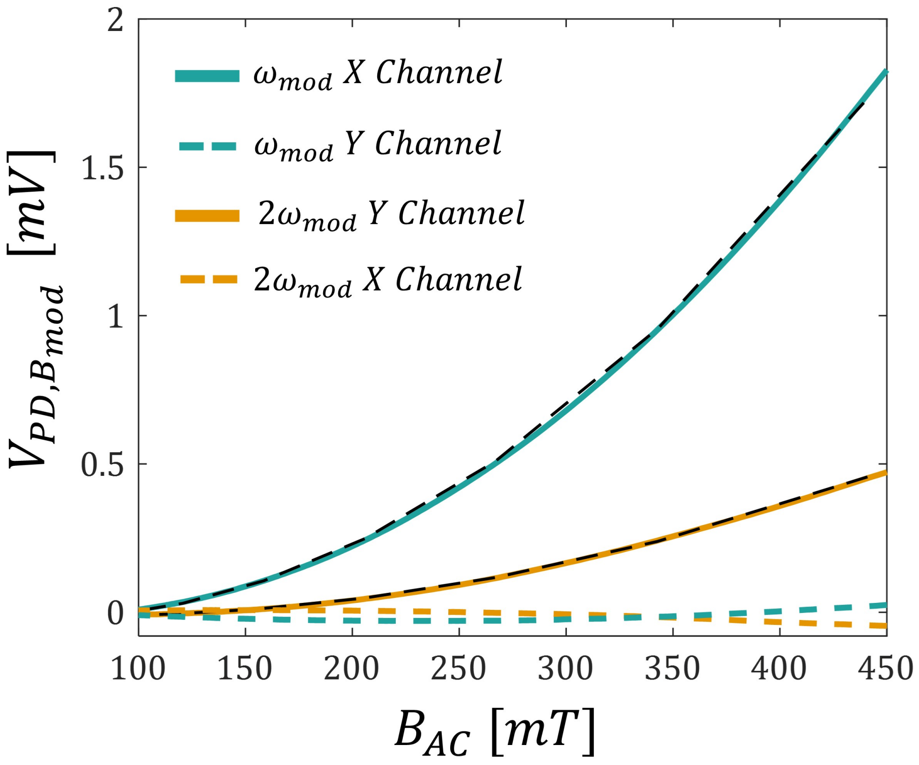


**Fig. S2. Measured response of Py at the fundamental and second harmonics. Green lines represent the measured signal at $\omega_{mod}$. Yellow lines represent the measured signal at $2\omega_{mod}$. Black dashed lines represent a fit to a quadratic function.**

## Supplementary Note 3: Deviations from ideal cross-polarization

To determine the relation between $V_{PD}$ and $B$, we return to Eq. (1) describing $V_{PD}$ in the case of an analyzer that is rotated by an angle $\phi$ from perfect extinction. In Eq. (1), the first term is the Fresnel reflection and is independent of $B$, the second term stems purely from the Kerr effect and depends on $B^2$. The third term stems from an intermixing of the primary $s$-polarized beam and the Kerr response and depends linearly on $B$. $|r_{ss}|^2 \gg |r_{ps}|^2$ by a factor of $\sim 10^6 - 10^7$ for typical ferromagnets, e.g. permalloy (Py). Therefore, for small nonvanishing $\phi$, the first and third terms can dominate and modify the relationship between $V_{PD}$ and $B$.

$V_{PD}$ is the demodulated signal measured by the lock-in amplifier, therefore, $V_{PD}$ will depend on the applied modulation scheme. The two cases of $B_{mod}$ and $L_{mod}$ appearing in the manuscript are described below:

**1) $\boldsymbol{B}$-modulation**:

When $B$-modulation is applied, only $r_{ps}$ is modulated such that the second and third terms of Eq. (1) will be measured by the lock-in amplifier. Consequently, $V_{PD,B_{mod}} = \frac{1}{2} \cdot c\epsilon_0\rho_0 \cdot \left|E_s^i\right|^2 \cdot \left[\cos^2\phi \left|r_{ps}\right|^2 + 2\cos\phi\sin\phi \cdot Re\{r_{ss}r_{ps}^*\}\right]$. Figure S3(a) shows the calculated $V_{PD,B_{mod}}$ for small $\phi$ angles in the range $0° - 0.06°$. To show that $V_{PD,B_{mod}}$ becomes linear with $B$, in Fig. S3(b) we present the same traces while normalizing each trace to its maximal value. It is readily seen that $V_{PD}$ becomes nearly linear with $B$ already at $\phi = 0.06°$. This behavior was also confirmed experimentally as shown in Fig. S3(c) which presents the $\phi$-dependent $V_{PD,B_{mod}}$ measurements for the Py sample. The small $\phi$ angles of the experiment were achieved using a standard precision rotational mount. Figure S3(d) presents the measured data after normalization revealing once more the transformation to a linear dependence. Overall, the measurements agree well with the calculated traces.

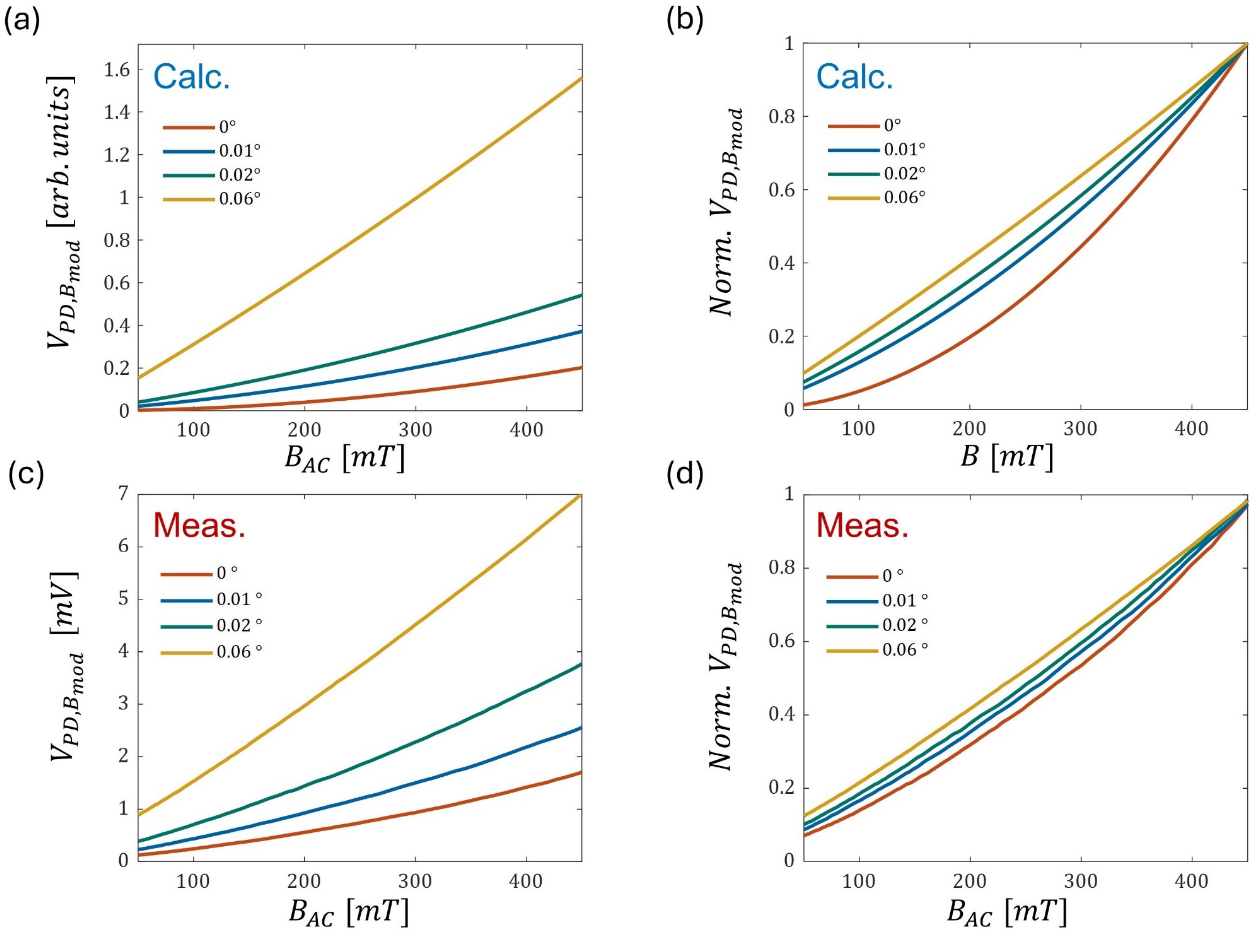


**Fig. S3. Calculated and measured $V_{PD}$ for $B$-modulation and various small $\phi$ angles. (a) Calculated $V_{PD,B_{mod}}$ for $\phi = 0°, 0.01°, 0.02°$, and $0.06°$. (b) Calculated $V_{PD,B_{mod}}$ presented in normalized units. Each trace was normalized to unity to illustrate the transition from a parabolic a linear dependence on $B$. (c) Measured $V_{PD,B_{mod}}$ in Py for $\phi = 0°, 0.01°, 0.02°$, and $0.06°$. (d) Measured $V_{PD,B_{mod}}$ presented in normalized units.**

**<u>2) Light-modulation</u>**:

In the $L$-modulation case, $\left|E_s^i\right|^2$ is modulated. Therefore, $V_{PD}$ accounts for all terms of Eq. (1):

$$V_{PD,L_{mod}} = \frac{1}{2} \cdot c\epsilon_0\rho_0 \cdot \left|E_s^i\right|^2 \cdot \left[\sin^2\phi\,|r_{ss}|^2 + \cos^2\phi\,\left|r_{ps}\right|^2 + 2\cos\phi\sin\phi \cdot Re\{r_{ss}r_{ps}^*\}\right]$$

As compared to the $B$-modulation case, the additional $\sin^2\phi\,|r_{ss}|^2$ term adds a constant $B$-independent shift to $V_{PD}$ which increases rapidly with $\phi$ due to the relatively large

magnitude of $|r_{ss}|^2$. Figure S4 presents the calculated and measured $\phi$-dependent $V_{PD,L_{mod}}$. The shift is readily seen in the calculated responses of Fig. S4(a). The normalized responses are presented in Fig. S4(b) and illustrate once more the transition from a parabolic to a linear dependence on $B$. The corresponding measurements in Py are presented in Fig. S4(c) and reproduce the shift in $V_{PD}$. The normalized measured responses of Fig. S4(d) illustrate the transition to a linear dependence on $B$ when $\phi \neq 0°$. As compared to the measured traces using $B$-modulation of Fig. S3, it is seen that the $L_{mod}$ measurements are noisier.

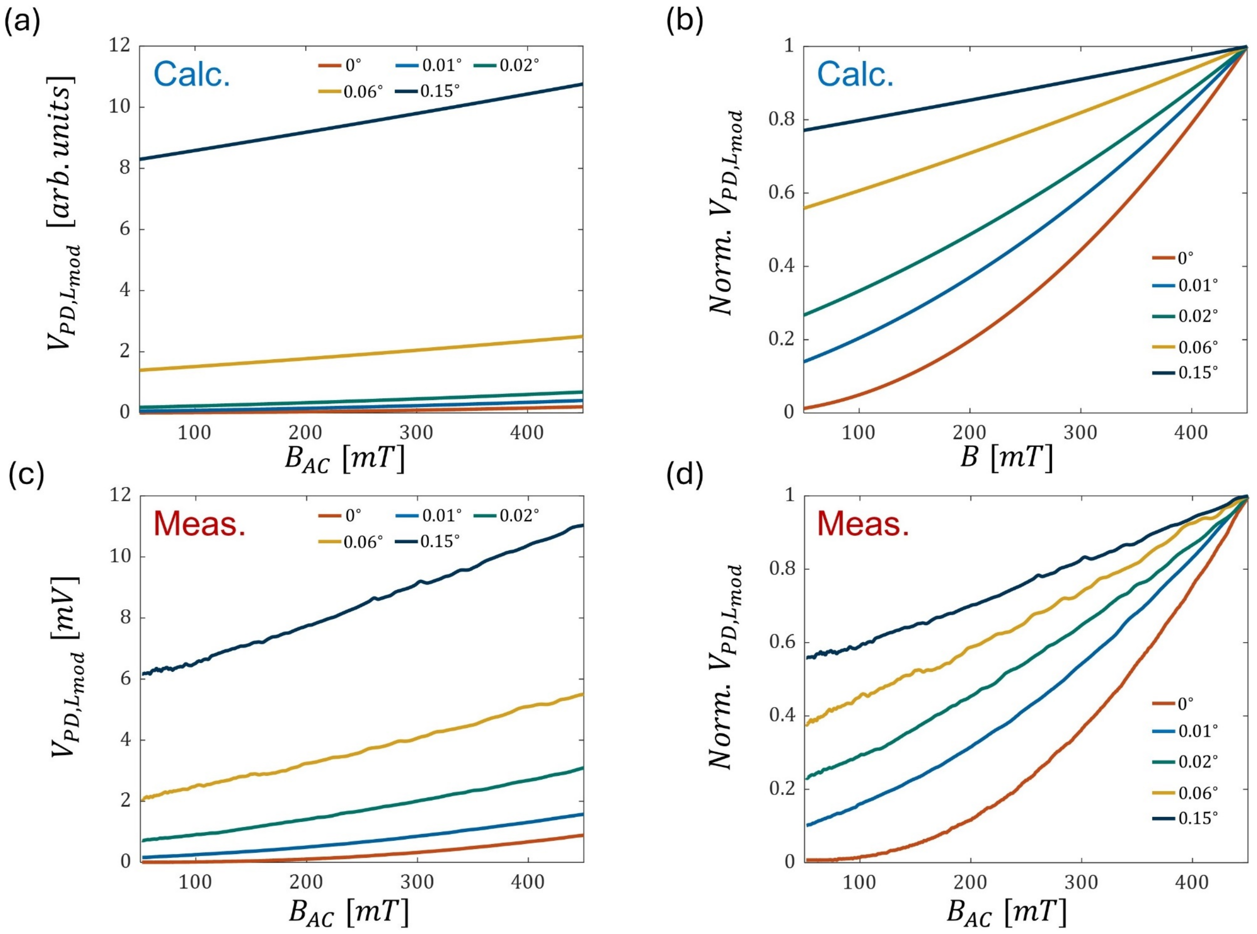


**Fig. S4. Calculated and measured $V_{PD}$ for $L$-modulation and various small $\phi$ angles. (a) Calculated $V_{PD,L_{mod}}$ for $\phi = 0°, 0.01°, 0.02°$, and $0.06°$. (b) Calculated $V_{PD,L_{mod}}$ presented in normalized units. Each trace was normalized to the maximal value to illustrate the transition from a parabolic a linear dependence on $B$. (c) Measured $V_{PD,L_{mod}}$ in Py for $\phi = 0°, 0.01°, 0.02°$, and $0.06°$. (d) Measured $V_{PD,L_{mod}}$ presented in normalized units.**

## Supplementary Note 4: Detection using a balanced detector

Another common implementation of the detection line utilizes a balanced detector [S6,7]. In this implementation, the reflected beam is rotated by $45°$ and is passed through a Wollaston prism which splits the beam to the two ports of the balanced detector. The voltage on at each port results from both the $s$ and $p$ components of the polarization and is given by [S1]:

$$\begin{bmatrix} V_1 \\ V_2 \end{bmatrix} \propto \frac{1}{2}\left|E_s^i\right|^2 \begin{bmatrix} |r_{ss}|^2 + \left|r_{ps}\right|^2 + 2Re\{r_{ss}r_{ps}^*\} \\ |r_{ss}|^2 + \left|r_{ps}\right|^2 - 2Re\{r_{ss}r_{ps}^*\} \end{bmatrix}.$$

The MOKE response is then extracted from the difference signal $V_1 - V_2$:

$$V_{diff} \propto \left|E_s^i\right|^2 \cdot Re\{r_{ss}r_{ps}^*\}.$$

It is readily seen that $V_{diff} \propto r_{sp}$ which linearly depends on $B$. This detection scheme inherently eliminates the purely parabolic MOKE signal $\left|r_{ps}\right|^2$ always resulting in a linear relationship with $B$.

Interestingly, from the expression for $V_{diff}$, it is seen that when the balanced detection is used, the $B$-modulation is not necessarily advantageous over the $L$-modulation scheme since both modulate $V_{diff}$ in the same fashion: the two quadratic terms were already eliminated when taking the difference $V_1 - V_2$.

This detection line can be used to extract $\theta_k$ and $\epsilon_k$ by including a controllable phase retarder that introduces the phase retardation, $\gamma$. In this case $V_{diff} \propto [\cos(\gamma)\theta_k + \sin(\gamma)\varepsilon_k]$. Namely, two measurements having $\gamma$ of $0°$ and $90°$ are required.

## Supplementary Note 5: XRD, AFM, and resistivity characterization

### X-ray diffraction measurements

X-ray diffraction (XRD) measurements were performed on thin films of Pt, Ta, Al, Au, and Cu that were grown on undoped 2-inch (100) Si/$SiO_2$ substrates. The substrates were $500\ \mu m$ thick and had a thermally oxidated $SiO_2$ layer of $25\ nm$. The Si substrates were single sided polished, backside etched and had a surface roughness smaller than $0.5\ nm$. The measurements were carried out using the D8 Advance diffractometer with LYNXEYE-XE-T detector (Bruker AXS, Karlsruhe, Germany) operating in 1D mode. XRD patterns within the range of $10^{\circ}$ - $90^{\circ}\ 2\theta$ were recorded at room temperature using CuKα radiation ($\lambda = 1.5418$ Å) under the following conditions: tube voltage of $40\ kV$, tube current of $40\ mA$, step-scan mode with step size $0.02^{\circ}\ 2\theta$, and counting time of $0.5\ sec/step$. The unit-cell parameters and the crystal structure were obtained from Rietveld refinements using TOPAS Bruker software.

The measured XRD data is presented in Fig. S5. The contribution of the substrate is indicated by black dashed line. The peaks appearing at $33^{\circ}$ and $70^{\circ}$ (marked by black arrows) corresponds to the lines of Si (211) and Si (400), respectively. Arrows marked in the Pt, Al, Au, and Cu, spectra indicate a preferred (111) crystal orientation. Arrows in the XRD data of Ta correspond to the $\beta$ crystalline phase as expected in room-temperature growth conditions. The extracted lattice parameters of Pt, Al, Au, and Cu, were 3.92 Å, 3.784 Å, 3.591 Å, and 3.608 Å, respectively, and the a and c lattice parameters of Ta were 10.194 Å and 5.31 Å, respectively. These lattice parameters are in good agreement with tabulated data.

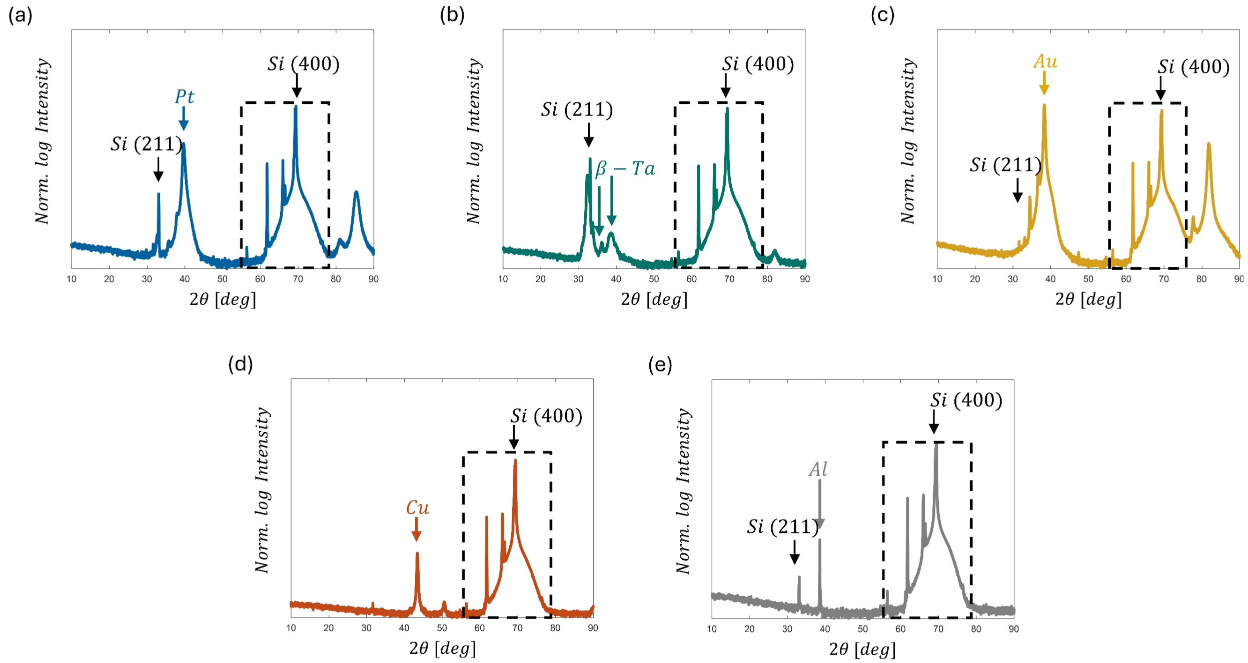


**Fig. S5. XRD characterization of the $50\ nm$ films of (a) Pt. (b) Ta. (c) Au. (d) Cu. and (e) Al. Colored arrows indicate the (111) crystal orientation in the Pt, Al, Au, and Cu spectra. Dashed lines indicate the contribution stemming from the substrate where the black arrows correspond to Si (400) and Si (211) lines. Arrows in Ta data corresponds to the $\beta$ phase of Ta.**

## Atomic force microscopy measurements

Figure S6 presents atomic force microscopy (AFM) data. The measurements were performed using the "Dimension Icon XR" (Extreme Research System) AFM system. All films were measured under the same configuration. The RMS surface roughness values, $R_q$, of the samples were $R_{q,Pt} = 0.384\,nm$, $R_{q,Ta} = 0.27\,nm$, $R_{q,Al} = 3.5\,nm$, $R_{q,Au} = 0.55\,nm$, and $R_{q,Cu} = 0.71\,nm$ indicating high-quality growth.

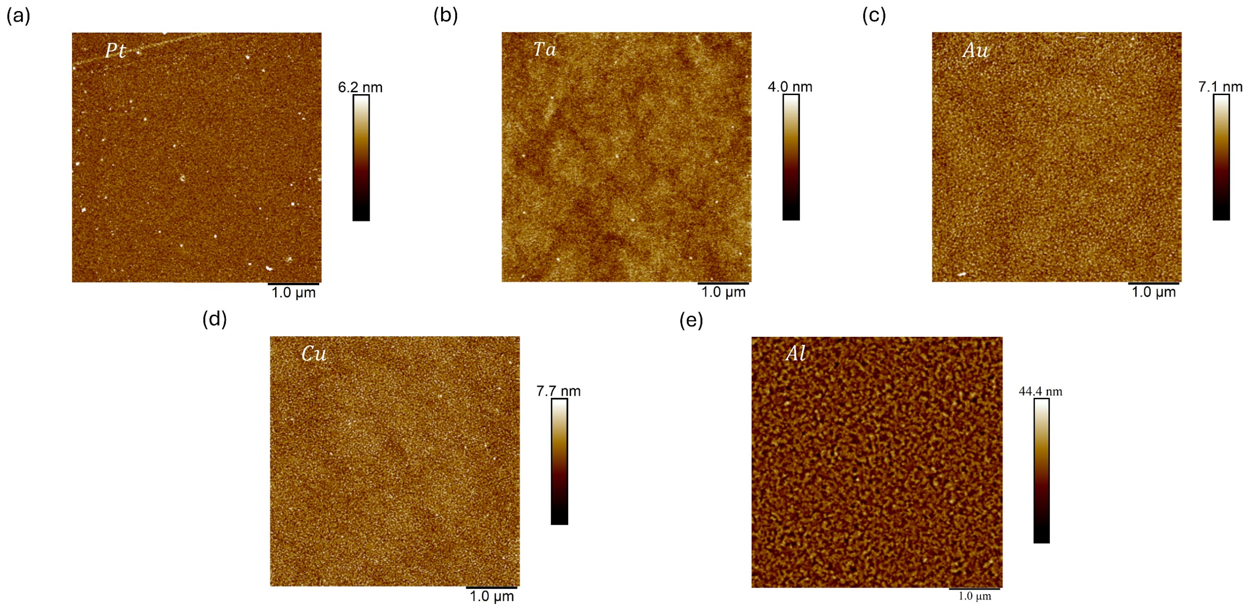


**Fig. S6. Surface morphology characterization. AFM measurements of (a) Pt. (b) Ta. (c) Au. (d) Cu. and (e) Al. The $R_q$ values were $R_{q,Pt} = 0.384\,nm$, $R_{q,Ta} = 0.27\,nm$, $R_{q,Au} = 0.55\,nm$, $R_{q,Cu} = 0.71\,nm$, and $R_{q,Al} = 3.5\,nm$.**

## Resistivity measurements

The resistivity of the samples was extracted from the DC Hall measurements which were carried out on dies of $10 \times 10\, mm^2$ in the Van der Pauw configuration. The resistivity values are summarized in Table S2 together with a comparison to values reported in the literature. The measured values are consistent with the previous reports. The measured resistivity of Ta corresponds to the $\beta$-phase and further support the conclusion that $\beta$-Ta was grown.

| Material | Hall measurements $[\mu\Omega \cdot cm]$ | Ref. A $[\mu\Omega \cdot cm]$ | Ref. B $[\mu\Omega \cdot cm]$ |
|---|---|---|---|
| Pt | $19.2$ | $20$ Ref. [S8] | $20.5$ Ref. [S9] |
| Ta | $173$ | $172$ Ref. [S10] | $170$ Ref. [S11] |
| Al | $24.1$ | $27.6$ Ref. [S12] | $30.0$ Ref. [S13] |
| Au | $3.9$ | $5.8$ Ref. [S14] | $3.4$ Ref. [S15] |
| Cu | $3.3$ | $2.75$ Ref. [S16] | $3.09$ Ref. [S2,17] |

**Table S2. Resistivity extracted from DC Hall transport measurements.**

## Supplementary Note 6: Shot noise analysis with non-ideal polarizers

In order to quantify the shot-noise, the extinction ratio ($ER$) of the polarizers should be taken into account. Following a similar analysis by Jin et. al [S18], the Jones matrix for a non-ideal analyzer placed at an angle $\phi$ is given by:

$$A_{n.i} = \begin{bmatrix} \cos^2\phi + \left(\sqrt{ER}\right)^{-1}\sin^2\phi & \cos\phi\sin\phi - \left(\sqrt{ER}\right)^{-1}\cos\phi\sin\phi \\ \cos\phi\sin\phi - \left(\sqrt{ER}\right)^{-1}\cos\phi\sin\phi & \sin^2\phi + \left(\sqrt{ER}\right)^{-1}\cos^2\phi \end{bmatrix}.$$

Following the derivation appearing in "Supplementary Information Note 1":

$$\begin{bmatrix} E_p^f \\ E_s^f \end{bmatrix} = \begin{bmatrix} r_{ps}E_0\left(\cos^2\phi + \left(\sqrt{ER}\right)^{-1}\sin^2\phi\right) + r_{ss}E_0\cos\phi\sin\phi\left(1-\left(\sqrt{ER}\right)^{-1}\right) \\ r_{ps}E_0\cos\phi\sin\phi\left(1-\left(\sqrt{ER}\right)^{-1}\right) + r_{ss}E_0\left(\sin^2\phi + \left(\sqrt{ER}\right)^{-1}\cos^2\phi\right) \end{bmatrix}.$$

For small $\phi$, the optical intensity $I_f$ reaching the detector can be described by $I_f = I_0|r_{ss}|^2\left(\phi^2 + 2\phi\frac{|r_{ps}|}{|r_{ss}|} + \left(\frac{|r_{ps}|}{|r_{ss}|}\right)^2 + \frac{1}{ER}\right)$, where $I_0$ is the initial intensity of the laser. The shot noise current is given by $I_{NS} = \sqrt{2\cdot q\cdot BW_{det}\cdot I_{det}}$, where $BW_{det}$ is the bandwidth of the photodetector which is $10\,MHz$ in our case, and $I_{det} = \eta_{det}I_f$ where, $\eta_{det}$ is the responsivity of the detector. Table S3 presents the calculated shot noise current together with the detected photocurrent stemming from the quadratic term $I_{quad} = \eta_{det}\cdot I_0\left(\frac{|r_{ps}|}{|r_{ss}|}\right)^2$ and the linear term $I_{linear} = \eta_{det}\cdot I_0 2\phi\frac{|r_{ps}|}{|r_{ss}|}$. The currents are calculated for a set of $\phi$ and $ER$ values and representative values of $r_{sp}$ and $r_{ss}$ of FMs and NMs. It is seen that the quadratic term is detectable in FMs for $ER > 3\cdot 10^4$. In contrast, detection of the quadratic term in NMs is possible only at maximal extinction ($\phi = 0°$) and high $ER$.

| $\phi$ $[deg]$ | Material | Extinction ratio | $I_{NS}$ $[nA]$ | $I_{quad}$ $[nA]$ | $I_{linear}$ $[nA]$ |
|---|---|---|---|---|---|
| $0°$ | FM | $3\cdot10^4$ | $4.61$ | $97.4$ | - |
| | | $1\cdot10^5$ | $2.52$ | $97.4$ | - |
| | | $1\cdot10^6$ | $0.8$ | $97.4$ | - |
| | NM | $3\cdot10^4$ | $4.61$ | $0.974$ | - |
| | | $1\cdot10^5$ | $2.52$ | $0.974$ | - |
| | | $1\cdot10^6$ | $0.8$ | $0.974$ | - |
| $0.06°$ | FM | $3\cdot10^4$ | $4.79$ | $97.4$ | $292.5$ |
| | | $1\cdot10^5$ | $2.83$ | $97.4$ | $292.5$ |
| | | $1\cdot10^6$ | $1.50$ | $97.4$ | $292.5$ |
| | NM | $3\cdot10^4$ | $4.72$ | $0.974$ | $29.23$ |
| | | $1\cdot10^5$ | $2.68$ | $0.974$ | $29.23$ |
| | | $1\cdot10^6$ | $0.94$ | $0.974$ | $29.23$ |
| $0.6°$ | FM | $3\cdot10^4$ | $10.04$ | $97.4$ | $2923.6$ |
| | | $1\cdot10^5$ | $9.2$ | $97.4$ | $2923.6$ |
| | | $1\cdot10^6$ | $8.95$ | $97.4$ | $2923.6$ |
| | NM | $3\cdot10^4$ | $9.61$ | $0.974$ | $292.46$ |
| | | $1\cdot10^5$ | $8.80$ | $0.974$ | $292.46$ |
| | | $1\cdot10^6$ | $8.47$ | $0.974$ | $292.46$ |

**Table S3. Comparison of shot noise and quadratic and linear terms of Eq. (1) of the manuscript. The table presents data for $\frac{|r_{sp}|}{|r_{ss}|}|_{0.5T}$ of $6.98\cdot10^{-4}$ and $6.98\cdot10^{-5}$ corresponding to typical values in FMs and NMs, respectively. Red color coding indicates signals that are smaller than the shot noise and green indicates higher values.**

## Supplementary Note 7: Considerations for measuring weak noise signals

The maximal extinction polarizer-analyzer arrangement is beneficial for weak noise measurements. Measuring the noise in the linear regime by rotating the analyzer away from maximal extinction, imposes the following difficulties.

In the linear regime, the detection shot noise increases significantly as shown in Note 4. The intense primary polarization and intermixing components will reach the detector and increase the shot noise level.

Furthermore, in the linear regime, the detection shot noise induced by the significant linear term may affect the signal recorded by the lock-in amplifier. In this regime, the strong linear intermixing term will reach the detector. It contributes to the shot noise according to: $I_{NS} \approx \sqrt{2 \cdot q \cdot BW_{det} \cdot I_0 \cdot \left(|r_{ss}|^2\phi^2 + |r_{ps}||r_{ss}|\phi + \frac{|r_{ss}|^2}{ER}\right)}$. Since $r_{ps}$ is modulated by $B$, the intermixing shot noise term will also be modulated and may contaminate the signal recorded by the lock-in amplifier.

Finally, it is well known that the resolution of the measurement decreases as the magnitude of the measured signal increases. Since the noise seems to be independent of the magnetic field, it is understood to be additive to the magneto optic signal and independent of it. Therefore, in the linear regime, where magneto-optic signal is much stronger, the measured voltages are orders of magnitude higher and the sensitivity to the small fluctuations is significantly reduced. Actually, even if the noise depends on the magneto optic effect, it would be difficult to study the noise in the linear regime since the noise statistics in this case are influenced additionally by the cross-correlation between $r_{ps}$ and $r_{ss}$ whose nature is unknown. The cross-correlation stems from the intermixing term. In the quadratic regime, the pure $r_{ps}$ term is measured and only the non-vanishing second moment contributes to the noise.

## Supplementary Note 8: Gilbert damping measurements

$\alpha$ measurements in Py-based bilayers were carried out using an optically probed spin-torque ferromagnetic resonance technique (OSTFMR) as described in Refs. [S19-21]. The experimental setup consists of a Ti:Sapphire emitting $35\ fs$ pulses at $800\ nm$ with a spot size of $\sim 10\ \mu m$. The laser was phased locked to a radio frequency (RF) signal which was in the range of $f = 6 - 13\ GHz$. The external magnetic field $B$ was applied in the sample plane using an electromagnet. The out-of-plane component of the AC magnetization, $m_z$, was probed using the magneto-optical Kerr effect (MOKE) in cross-polarization configuration. A delay line was integrated into the optical beam path and provided temporal resolution.

In these experiments, new samples were fabricated by magnetron sputtering as described in the "Methods" section of the manuscript. The samples were patterned into $25 \times 55\ \mu m^2$ $(W \times L)$ devices using lift-off lithography. The RF signal was driven through the sample as required in the OSTFMR technique. The bi-layer films consisted of $25\ X\ |\ 10\ Py\ |\ 2\ TaN$ (units in $nm$) where $X$ = Pt, Ta, Al, Au, and Cu. The TaN layer was added as a capping layer to prevent oxidation of the Py layer. An additional sample consisting of a single layer of Py was fabricated to measure the intrinsic damping of Py. This sample consisted of $2\ TaN|\ 10\ Py\ |\ 2\ TaN$ where the first layer TaN served as the seeding layer.

The $m_z$ spectra consist of the in-phase and out-of-phase components. Therefore, to extract the resonance field, $H_{res}$, and linewidth, $\Delta H$, the $m_z$ spectra were fitted to a complex Lorentzian function $\chi(B) = Re\left\{\frac{A \cdot e^{i\varphi}}{(\mu_0^2 H_{res}^2 - B^2) + i\mu_0 \Delta H B}\right\}$ using a non-linear least square algorithm. $A$ is the amplitude of the Lorentzian function, $\varphi$ is the phase, and $\mu_0$ is the vacuum permeability. The saturation magnetization, $M_s$, was extracted using Kittel's formula, $f = \gamma\mu_0/2\pi\sqrt{H_{res}(H_{res} + M_s)}$, where $\gamma$ is the gyromagnetic ratio.

Figures S7 - S12 present the measured FMR data for all samples. Panels (a) in the figures present the measured spectra (blue solid line) together with the corresponding fits to the complex Lorentzian functions (solid orange line). Panels (b) present the $f - H_{res}$

dispersion curves from which $M_s$ was extracted. Panels (c) present the $\Delta H - f$ relations from which $\alpha_{NM/Py}$ was extracted.

The measured $M_s$ were $6.79 \times 10^5\ A/m$, $6.85 \times 10^5\ A/m$, $6.36 \times 10^5\ A/m$, $6.62 \times 10^5\ A/m$, and $6.73 \times 10^5\ A/m$ for the Pt, Ta, Al, Au, and Cu based bilayers, respectively, and $M_s$ of the additional Py sample was $6.89 \times 10^5\ A/m$. $\alpha$ was extracted from the $\Delta H - f$ relationship, $\mu_0 \Delta H = f \cdot 4\pi\alpha/\gamma + \mu_0 \Delta H_{IH}$, where $\Delta H_{IH}$ is the inhomogeneous linewidth broadening. The measured $\alpha_{NM/Py}$ of the bilayers were $11.9 \times 10^{-3}$, $9.5 \times 10^{-3}$, $8.3 \times 10^{-3}$, $7.7 \times 10^{-3}$, and $6.7 \times 10^{-3}$ for Pt, Ta, Al, Au, and Cu, respectively, and $\alpha_{Py}$ was measured to be $5.6 \times 10^{-3}$. The $\alpha$ enhancement was calculated by $\alpha_{sp}^{Py} = \alpha_{NM/Py} - \alpha_{Py}$. Table S4 summarizes the data extracted from the OSTFMR measurements.

| **Sample** | $M_S$ $[\times 10^5\ A/m]$ | $\alpha$ $[\times 10^{-3}]$ | $\alpha_{sp}^{Py}$ $[\times 10^{-3}]$ |
|---|---|---|---|
| **Pt/Py** | **6.79** | **11.9** | **6.3** |
| **Ta/Py** | **6.85** | **9.5** | **3.9** |
| **Al/Py** | **6.36** | **8.3** | **2.7** |
| **Au/Py** | **6.62** | **7.7** | **2.1** |
| **Cu/Py** | **6.73** | **6.7** | **1.1** |
| **Py** | **6.89** | **5.6** | **NA** |

**Table S4. $M_s$, $\alpha$, and $\alpha_{sp}^{Py}$ extracted from dynamical OSTFMR measurements.**

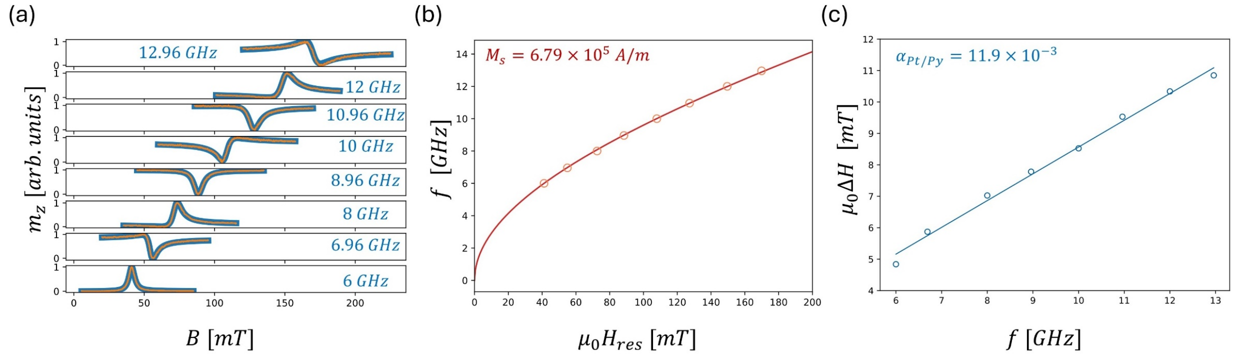


**Fig. S7. OSTFMR measurement of Pt. (a) Measured FMR (blue) and fit (orange). (b) $f - H_{res}$ dispersion relations. Open circles represent measured data and the solid line represents the fit to Kittel's formula. (c) $\Delta H$ vs. $f$. Open circles represent measured data and the solid line represents the linear fit.**

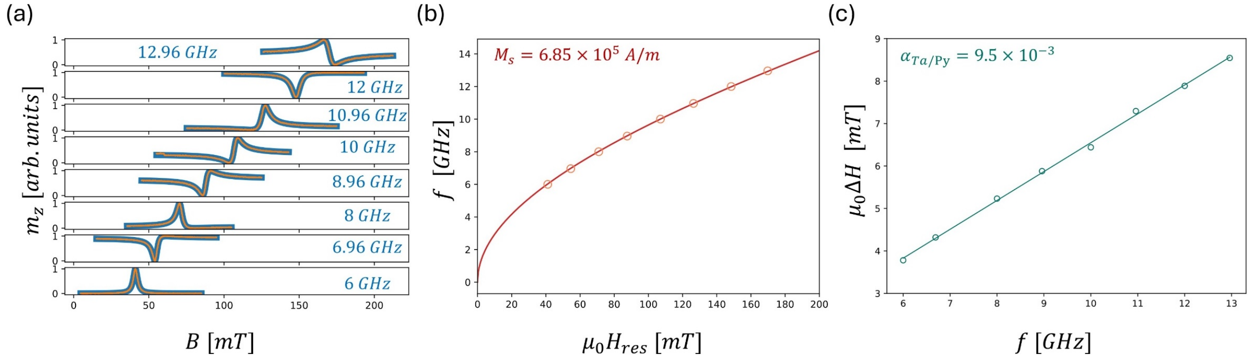


**Fig. S8. OSTFMR measurement of Ta. (a) Measured FMR (blue) and fit (orange). (b) $f - H_{res}$ dispersion relations. Open circles represent measured data and the solid line represents the fit to Kittel's formula. (c) $\Delta H$ vs. $f$. Open circles represent measured data and the solid line represents the linear fit.**

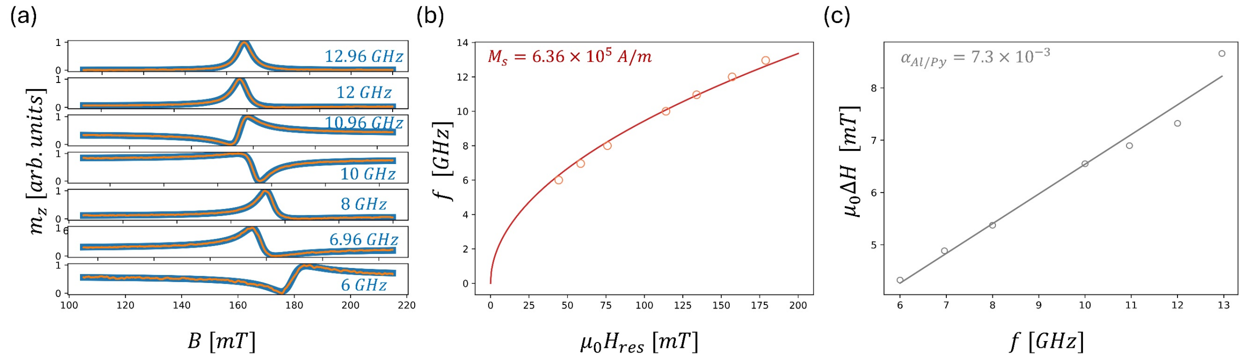


**Fig. S9. OSTFMR measurement of Al. (a) Measured FMR (blue) and fit (orange). (b) $f - H_{res}$ dispersion relations. Open circles represent measured data and the solid line represents the fit to Kittel's formula. (c) $\Delta H$ vs. $f$. Open circles represent measured data and the solid line represents the linear fit.**

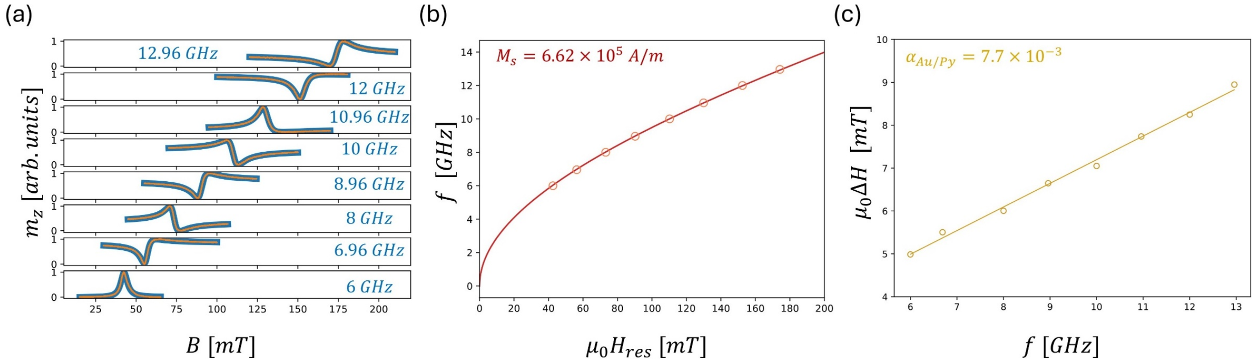


**Fig. S10. OSTFMR measurement of Au. (a) Measured FMR (blue) and fit (orange). (b) $f - H_{res}$ dispersion relations. Open circles represent measured data and the solid line represents the fit to Kittel's formula. (c) $\Delta H$ vs. $f$. Open circles represent measured data and the solid line represents the linear fit.**

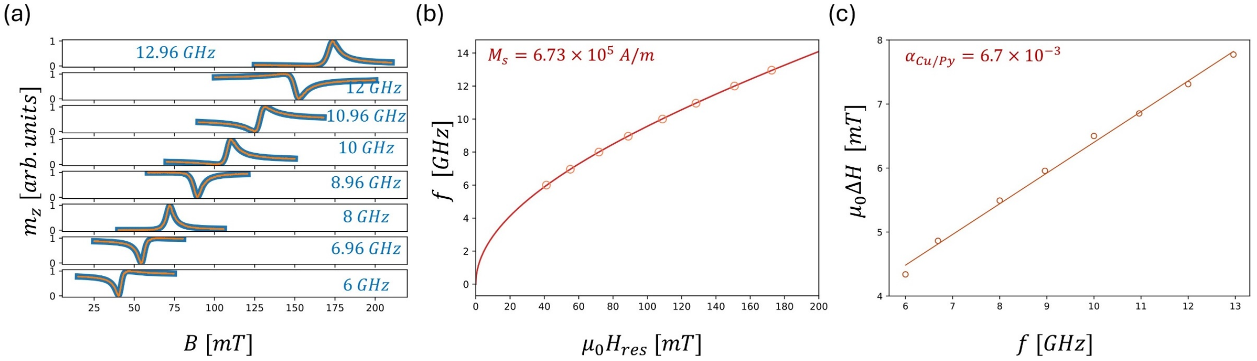


**Fig. S11. OSTFMR measurement of Cu. (a) Measured FMR (blue) and fit (orange). (b) $f - H_{res}$ dispersion relations. Open circles represent measured data and the solid line represents the fit to Kittel's formula. (c) $\Delta H$ vs. $f$. Open circles represent measured data and the solid line represents the linear fit.**

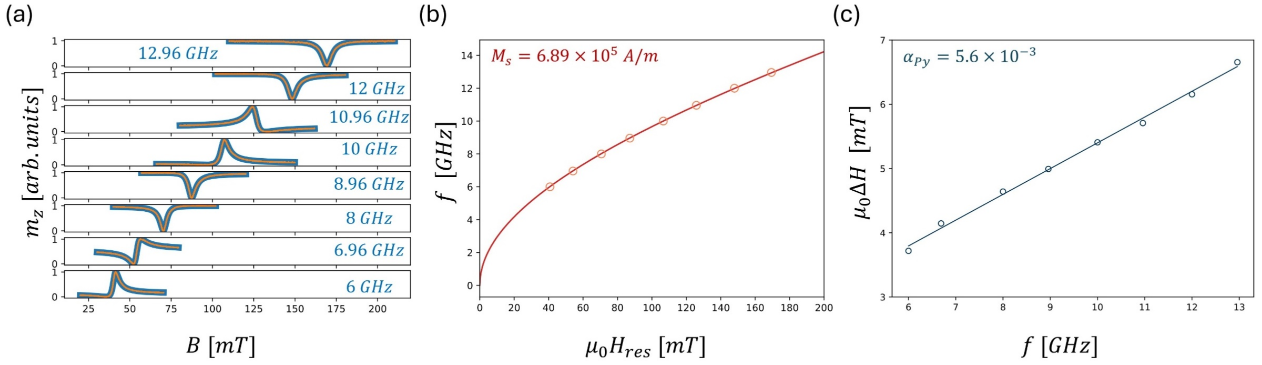


**Fig. S12. OSTFMR measurement of Py. (a) Measured FMR (blue) and fit (orange). (b) $f - H_{res}$ dispersion relations. Open circles represent measured data and the solid line represents the fit to Kittel's formula. (c) $\Delta H$ vs. $f$. Open circles represent measured data and the solid line represents the linear fit.**

## Supplementary Note 9: Measurements at $638\ nm$

The measurements were repeated using a red $638\ nm$ laser (Cobolt 06-MLD, 10 $mW$, beam size: $700 \times 700\ \mu m^2$) which can shed light on the role of interband transitions. Figure S13(a) presents the measured $V_{PD,B_{mod}}$, exhibiting a clear quadratic trend. At this wavelength, the response is strongest for Au, followed by Cu, Ta, Pt, and then Al, suggesting distinct spectral features in the range of $1.9 - 2.8\ eV$. Figure S13(b) presents the measured $|\Phi_K|$ data illustrating a linear dependence on $B_{AC}$ for all metals. The $|\Phi_K|$ angles are overall consistent with the previously reported values [S22], as summarized in Table S5. The smaller $|\Phi_K|$ values may suggest smaller contributions from interband transitions. Figures. S13(c) and (d) present the noise analysis. Here, the lack of correlation between $\sqrt{\tilde{V}_{RMSE,norm}}$ and the optical Hall response is more evident. For instance, while Au exhibits the largest OHE response, it displays relatively low noise. In contrast, Pt displays a weak response accompanied by significant noise. Once again, as shown in Fig. S13(d), the noise scales with $\alpha_{sp}$ resulting in an R-square determination parameter of $0.915$.

| Sample | Present work | Ref. [S3], Uba et al. (2017)<br>Ref. [S2], Uba et al. (2000) | | Ref. [S4]<br>Schnatterly et al. (1969) | | Ref. [S5], Stern et al. (1964)<br>Ref. [S22], McGroddy et. al (1965) | |
|---|---|---|---|---|---|---|---|
| | Measured @ $0.45\ T$ $[mdeg]$ | Measured @ $1.5\ T$ $[mdeg]$ | Extrapolated to $0.45\ T$ $[mdeg]$ | Measured @ $1\ T$ $[mdeg]$ | Extrapolated to $0.45\ T$ $[mdeg]$ | Measured in units of $[mdeg/T]$ | Extrapolated to $0.45\ T$ $[mdeg]$ |
| Au | $\lvert\Phi_K\rvert = 0.36$ | $\lvert\Phi_K\rvert = 1.26$ | $\lvert\Phi_K\rvert = 0.42$ | $\lvert\Phi_K\rvert = 0.81$ | $\lvert\Phi_K\rvert = 0.36$ | $\theta_k = 0.8$ | $\theta_k = 0.36$ |
| Cu | $\lvert\Phi_K\rvert = 0.32$ | $\lvert\Phi_K\rvert = 0.61$ | $\lvert\Phi_K\rvert = 0.2$ | $\lvert\Phi_K\rvert = 0.28$ | $\lvert\Phi_K\rvert = 0.13$ | - | - |
| Pt | $\lvert\Phi_K\rvert = 0.18$ | $\lvert\Phi_K\rvert = 0.28$ | $\lvert\Phi_K\rvert = 0.085$ | - | - | - | - |
| Al | $\lvert\Phi_K\rvert = 0.17$ | - | - | - | - | $\theta_k = 0.31$ | $\theta_k = 0.15$ |
| Ta | $\lvert\Phi_K\rvert = 0.26$ | - | - | - | - | - | - |

**Table S5. Comparison of measured and previously reported $|\Phi_K|$ values at $638\ nm$.**

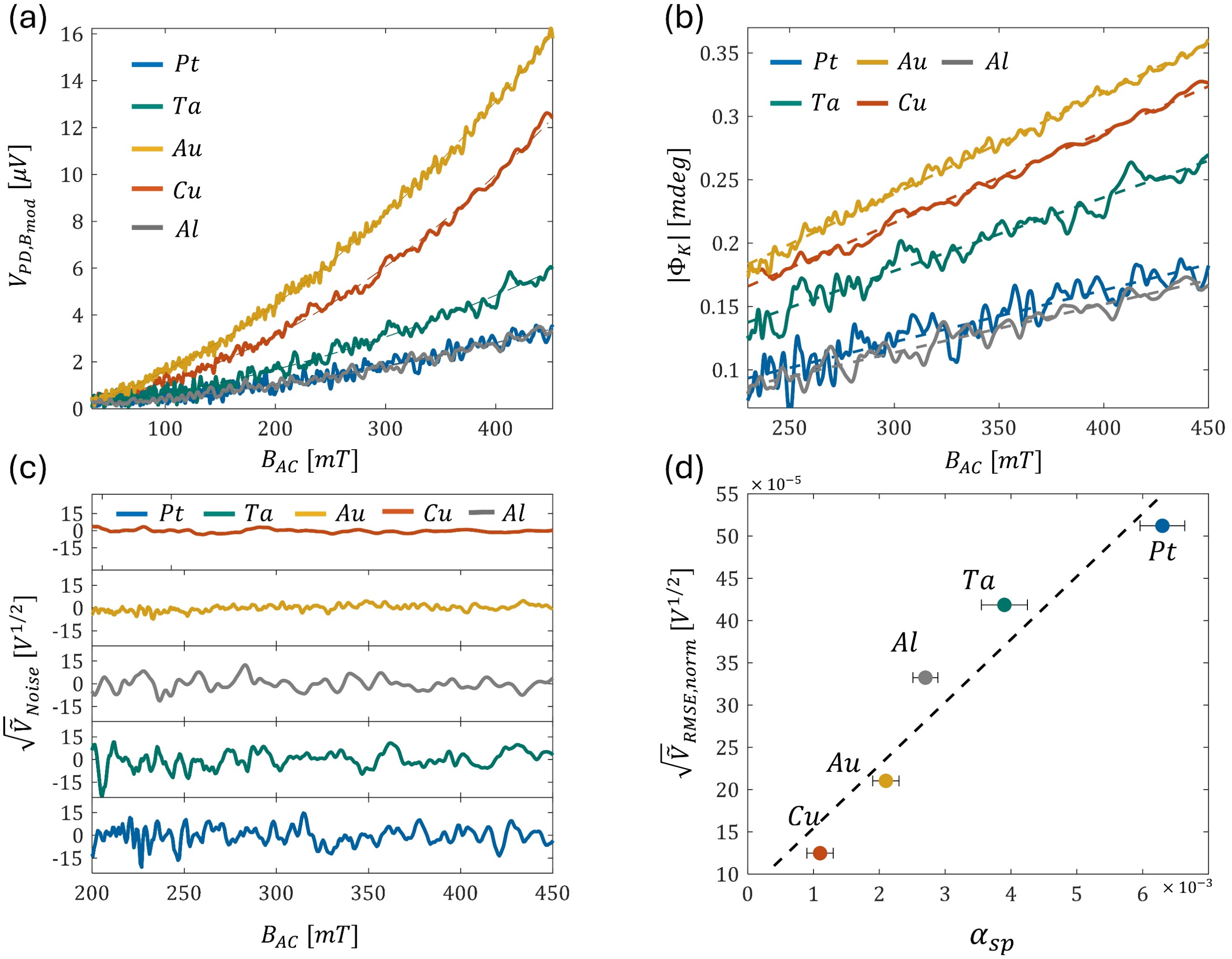


**Fig S13. (a) $V_{PD,B_{mod}}$ measured at $638\ nm$. (b) Extracted $|\Phi_K|$ values. (c) Extracted $\sqrt{\tilde{V}}_{Noise}$ traces as a function of $B_{AC}$. (d) $\sqrt{\tilde{V}}_{RMS,norm}$ as a function of $\alpha_{sp}^{Py}$.**

## References


[1] M. J. Gomez, K. Liu, J. G. Lee, and R. B. Wilson, ***"High sensitivity pump–probe measurements of magnetic, thermal, and acoustic phenomena with a spectrally tunable oscillator"***, Review of Scientific Instruments **91**, 023905 (2020).

[2] L. Uba, S. Uba, V. N. Antonov, A. N. Yaresko, and R. Gontarz, ***"Magneto-optical Kerr spectroscopy of platinum"***, Physical Review B **62**, 16510 (2000).

[3] L. Uba, S. Uba, and V. N. Antonov, ***"Magneto-optical Kerr spectroscopy of noble metals"***, Physical Review B **96**, 235132 (2017).

[4] S. E. Schnatterly, ***"Magnetoreflection Measurements on the Noble Metals"***, Physical Review **183**, 664 (1969).

[5] E. A. Stern, J. C. McGroddy, and W. E. Harte, ***"Polar Reflection Faraday Effect in Metals"***, Physical Review **135**, A1306 (1964).

[6] V. H. Ortiz, S. Coh, and R. B. Wilson, ***"Magneto-optical Kerr spectra of gold induced by spin accumulation"***, Physical Review B **106**, 014410 (2022).

[7] V. H. Ortiz, S. B. Mishra, L. Vuong, S. Coh, and R. B. Wilson, ***"Specular inverse Faraday effect in transition metals"***, Physical Review Materials **7**, 125202 (2023).

[8] S. Dutta, K. Sankaran, K. Moors, G. Pourtois, S. Van Elshocht, J. Bömmels, W. Vandervorst, Z. Tőkei, and C. Adelmann, ***"Thickness dependence of the resistivity of platinum-group metal thin films"***, Journal of Applied Physics **122**, 025107 (2017).

[9] J. S. Agustsson, U. B. Arnalds, A. S. Ingason, K. B. Gylfason, K. Johnsen, S. Olafsson, and J. T. Gudmundsson, ***"Growth, coalescence, and electrical resistivity of thin Pt films grown by dc magnetron sputtering on SiO2"***, Applied Surface Science **254**, 7356 (2008).

[10] E. A. I. Ellis, M. Chmielus, S. Han, and S. P. Baker, ***"Effect of sputter pressure on microstructure and properties of β-Ta thin films"***, Acta Materialia **183**, 504 (2020).

[11] G. Abadias, J. J. Colin, D. Tingaud, P. Djemia, L. Belliard, and C. Tromas, ***"Elastic properties of α- and β-tantalum thin films"***, Thin Solid Films **688**, 137403 (2019).

[12] G. P. Panta and D. P. Subedi, ***"Electrical characterization of aluminum (Al) thin films measured by using four- point probe method"***, Kathmandu University Journal of Science, Engineering and Technology **8**, 31 (2013).

[13] V. G, ***"Thickness dependence of the electrical properties of aluminum thin films"***, Journal of Emerging Technologies and Innovative Research **10**, no.b289 (2023).

[14] G. Kästle, H. G. Boyen, A. Schröder, A. Plettl, and P. Ziemann, ***"Size effect of the resistivity of thin epitaxial gold films"***, Physical Review B **70**, 165414 (2004).

[15] J. W. C. d. Vries, ***"Resistivity of thin Au films as a function of grain diameter and temperature"***, Journal of Physics F: Metal Physics **18**, 331 (1988).

[16] K. Barmak, X. Liu, A. Darbal, N. T. Nuhfer, D. Choi, T. Sun, A. P. Warren, K. R. Coffey, and M. F. Toney, ***"On twin density and resistivity of nanometric Cu thin films"***, Journal of Applied Physics **120**, 065106 (2016).

[17] K. Khojier and H. Savaloni, ***"A study on the dependence of DC electrical properties and nanostructure of Cu thin films on film thickness"***, International Journal of Nano Dimension **3**, 217 (2013).

[18] C. Jin, Z. Tao, K. Kang, K. Watanabe, T. Taniguchi, K. F. Mak, and J. Shan, ***"Imaging and control of critical fluctuations in two-dimensional magnets"***, Nature Materials **19**, 1290 (2020).

[19] B. Grover, B. K. Hazra, T. Ma, B. Pal, N. Bernstein, A. Rothschild, A. K. Srivastava, S. Choudhury, G. Woltersdorf, A. Capua, and S. S. P. Parkin, ***"Crystallographic dependence of the spin Hall angle in epitaxial Pt films: Comparison of optical and electrical detection of spin-torque ferromagnetic resonance techniques"***, Applied Physics Letters **120**, 172406 (2022).

[20] A. Capua, C. Rettner, and S. S. P. Parkin, ***"Parametric Harmonic Generation as a Probe of Unconstrained Spin Magnetization Precession in the Shallow Barrier Limit"***, Physical Review Letters **116**, 047204 (2016).
[21] A. Rothschild, N. Am-Shalom, N. Bernstein, M. y. Meron, T. David, B. Assouline, E. Frohlich, J. Xiao, B. Yan, and A. Capua, ***"Generation of spin currents by the orbital Hall effect in Cu and Al and their measurement by a Ferris-wheel ferromagnetic resonance technique at the wafer level"***, Physical Review B **106**, 144415 (2022).
[22] A. J. McAlister, E. A. Stern, and J. C. McGroddy, ***"Faraday Effect and Fermi Surfaces of the Silver-Gold Alloy System"***, Physical Review **140**, A2105 (1965).
